# *N*′-(2-Fluoro­benzo­yl)benzohydrazide

**DOI:** 10.1107/S1600536808014645

**Published:** 2008-05-21

**Authors:** Krzysztof Ejsmont, Muhammad Zareef, Muhammad Arfan, Sarfaraz A. Bashir, Jacek Zaleski

**Affiliations:** a Institute of Chemistry, University of Opole, Oleska 48, 45-052 Opole, Poland; b Department of Chemistry, Quaid-i-Azam University, Islamabad 45320, Pakistan; c Department of Environmental Sciences, International Islamic University, Islamabad 45320, Pakistan

## Abstract

In the crystal structure of the title compound, C_14_H_11_FN_2_O_2_, the molecule is centrosymmetric. The F atom is disordered over four positions, on the two ortho positions of each ring, with occupancies of 0.287:0.213 (5). In the crystal structure, mol­ecules are linked by inter­molecular N—H⋯O and C—H⋯O hydrogen bonds.

## Related literature

For related literature, see: Silva *et al.* (2006 ▶); Chopra *et al.* (2006 ▶); de Souza *et al.* (2007 ▶); Ahmad *et al.* (2001 ▶); Al-Soud, *et al.* (2004 ▶); Al-Talib *et al.* (1990 ▶); El-Emam *et al.* (2004 ▶); Yousif *et al.* (1986 ▶); Zareef & Iqbal (2007 ▶); Zheng *et al.* (2003 ▶).
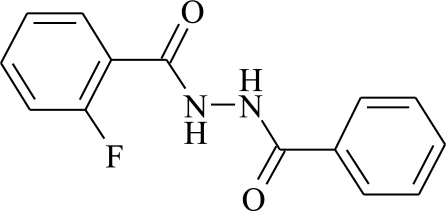


## Experimental

### 

#### Crystal data

C_14_H_11_FN_2_O_2_
*M* *_r_* = 258.25
Monoclinic, 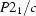

*a* = 4.7698 (10) Å
*b* = 5.2435 (10) Å
*c* = 23.913 (5) Å
β = 100.89 (3)°
*V* = 587.3 (2) Å^3^
*Z* = 2
Mo *K*α radiation
μ = 0.11 mm^−1^
*T* = 90.0 (1) K
0.25 × 0.20 × 0.10 mm

#### Data collection

Oxford Diffraction Xcalibur diffractometer
Absorption correction: none
3819 measured reflections
1205 independent reflections
1060 reflections with *I* > 2σ(*I*)
*R*_int_ = 0.044

#### Refinement

*R*[*F* ^2^ > 2σ(*F* ^2^)] = 0.034
*wR*(*F* ^2^) = 0.094
*S* = 1.07
1205 reflections
118 parameters
H atoms treated by a mixture of independent and constrained refinement
Δρ_max_ = 0.28 e Å^−3^
Δρ_min_ = −0.28 e Å^−3^

### 

Data collection: *CrysAlis CCD* (Oxford Diffraction, 2002 ▶); cell refinement: *CrysAlis RED* (Oxford Diffraction, 2006 ▶); data reduction: *CrysAlis RED*; program(s) used to solve structure: *SHELXS97* (Sheldrick, 2008 ▶); program(s) used to refine structure: *SHELXL97* (Sheldrick, 2008 ▶); molecular graphics: *SHELXTL* (Sheldrick, 2008 ▶); software used to prepare material for publication: *SHELXL97*.

## Supplementary Material

Crystal structure: contains datablocks global, I. DOI: 10.1107/S1600536808014645/bq2073sup1.cif

Structure factors: contains datablocks I. DOI: 10.1107/S1600536808014645/bq2073Isup2.hkl

Additional supplementary materials:  crystallographic information; 3D view; checkCIF report

## Figures and Tables

**Table 1:** Hydrogen-bond geometry (Å, °)

*D*—H⋯*A*	*D*—H	H⋯*A*	*D*⋯*A*	*D*—H⋯*A*
N1—H1⋯O3^i^	0.84 (2)	2.05 (2)	2.8549 (16)	160.4 (16)
N1—H1⋯O3^ii^	0.84 (2)	2.325 (16)	2.6302 (14)	101.8 (14)
C8—H8⋯O3^iii^	0.945 (17)	2.416 (16)	3.2687 (17)	150.0 (12)

Symmetry codes: (i) 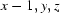; (ii) 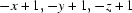; (iii) 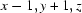.

**Fig. 1.**
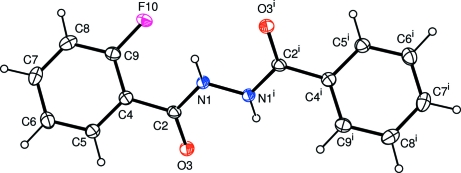
View of the title molecule with anisotropic displacement parameters shown at the 50% probability level. [Symmetry code: (i) -x, -y, -z].

**Fig. 2.**
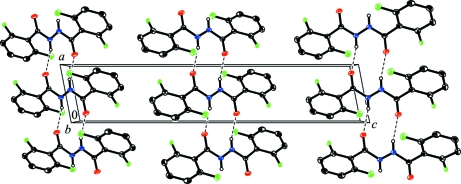
The packing diagram of the title compound. Dashed lines indicate hydrogen bonds.

## supplementary crystallographic information

### Comment

*N*,*N*'-Diacylhydrazines are very important intermediates especially for the synthesis of various biological active five member heterocyclic compounds such as 2,5-disubstituted-1,3,4-oxadiazoles (Zheng *et al.*, 2003; Al-Talib *et al.*, 1990) and 5-substituted-2-mercapto-1,3,4-oxadiazoles (Yousif *et al.*, 1986; Ahmad *et al.*, 2001; Al-Soud *et al.*, 2004; El-Emam *et al.*, 2004). In view of the versatility of these compounds, we have synthesized the title compound, (I), using a literature method (Zareef *et al.*, 2007) and reported its crystal structure. The geometry of (I) is normal and (Table 1) compares well with those found in other crystal structures (Silva *et al.*, 2006; Chopra *et al.*, 2006; Souza *et al.*, 2007). The title molecule, C_14_H_11_N_2_O_2_F, is non-planar. The dihedral angle between the benzene rings and CONHNHCO group is 34.5 (5) °. The disorder of the title molecule is realised by the presents of two positions for F atom with occupancy factors of 0.3 for F10 and 0.2 for F10'. The molecules are linked into a three-dimensional framework by a combination of two N–H···O and one weak C–H···O hydrogen bonds.

### Experimental

For the synthesis of title compound (I), benzoyl chloride (5.1 mmol) was added in portions to a suspension of 2-fluorobenzoic hydrazide (5.0 mmol) in dry acetonitrile (50 ml), and the reaction mixture was stirred for 9 h at 296 K. Then, the resulting mixture was concentrated, and the solid product filtered and recrystallized from aqueous ethanol to afford the title compound (yield; 87%). Suitable crystals were grown from a solution of (I) in ethanol by slow evaporation at room temperature.

### Refinement

The occupancy factors for the disordered fluorine and hydrogen (H5 and H9) atoms were refined using free variables. The H5 nad H9 were included in the refinement at geometrically idealized positions with C-H distances 0.96 A and their parameters are not refinement. The remaining H atoms were located in a difference map and freely refined.

### Crystal data

**Table:**

C_14_H_11_FN_2_O_2_	*F*(000) = 268
*M**_r_* = 258.25	*D*_x_ = 1.460 Mg m^−^^3^
Monoclinic, *P*2_1_/*c*	Mo *K*α radiation, λ = 0.71073 Å
Hall symbol: -P 2ybc	Cell parameters from 1205 reflections
*a* = 4.7698 (10) Å	θ = 3.5–26.5°
*b* = 5.2435 (10) Å	µ = 0.11 mm^−^^1^
*c* = 23.913 (5) Å	*T* = 90 K
β = 100.89 (3)°	Plate, colourless
*V* = 587.3 (2) Å^3^	0.25 × 0.20 × 0.10 mm
*Z* = 2	

### Data collection

**Table:**

Oxford Diffraction Xcalibur diffractometer	1060 reflections with *I* > 2σ(*I*)
Radiation source: fine-focus sealed tube	*R*_int_ = 0.044
graphite	θ_max_ = 26.5°, θ_min_ = 3.5°
Detector resolution: 1024 x 1024 with blocks 2 x 2 pixels mm^-1^	*h* = −5→5
ω scans	*k* = −4→6
3819 measured reflections	*l* = −30→30
1205 independent reflections	

### Refinement

**Table:**

Refinement on *F*^2^	Primary atom site location: structure-invariant direct methods
Least-squares matrix: full	Secondary atom site location: difference Fourier map
*R*[*F*^2^ > 2σ(*F*^2^)] = 0.034	Hydrogen site location: inferred from neighbouring sites
*wR*(*F*^2^) = 0.094	H atoms treated by a mixture of independent and constrained refinement
*S* = 1.07	*w* = 1/[σ^2^(*F*_o_^2^) + (0.0555*P*)^2^ + 1.578*P*] where *P* = (*F*_o_^2^ + 2*F*_c_^2^)/3
1205 reflections	(Δ/σ)_max_ < 0.001
118 parameters	Δρ_max_ = 0.28 e Å^−^^3^
0 restraints	Δρ_min_ = −0.28 e Å^−^^3^

### Special details

**Table:**

Geometry. All esds (except the esd in the dihedral angle between two l.s. planes) are estimated using the full covariance matrix. The cell esds are taken into account individually in the estimation of esds in distances, angles and torsion angles; correlations between esds in cell parameters are only used when they are defined by crystal symmetry. An approximate (isotropic) treatment of cell esds is used for estimating esds involving l.s. planes.
Refinement. Refinement of *F*^2^ against ALL reflections. The weighted *R*-factor *wR* and goodness of fit *S* are based on *F*^2^, conventional *R*-factors *R* are based on *F*, with *F* set to zero for negative *F*^2^. The threshold expression of *F*^2^ > σ(*F*^2^) is used only for calculating *R*-factors(gt) *etc*. and is not relevant to the choice of reflections for refinement. *R*-factors based on *F*^2^ are statistically about twice as large as those based on *F*, and *R*- factors based on ALL data will be even larger.

### Fractional atomic coordinates and isotropic or equivalent isotropic displacement parameters (Å^2^)

**Table:**

	*x*	*y*	*z*	*U*_iso_*/*U*_eq_	Occ. (<1)
N1	0.4363 (2)	0.5857 (2)	0.47949 (4)	0.0183 (3)	
C2	0.5853 (2)	0.6622 (2)	0.44055 (5)	0.0166 (3)	
O3	0.83154 (17)	0.58840 (19)	0.44180 (4)	0.0255 (3)	
C4	0.4404 (2)	0.8420 (2)	0.39624 (5)	0.0163 (3)	
C5	0.5167 (3)	0.8362 (2)	0.34288 (5)	0.0193 (3)	
H5	0.6561	0.7149	0.3356	0.023*	0.77 (6)
C6	0.3958 (3)	1.0022 (3)	0.30033 (5)	0.0228 (3)	
C7	0.1997 (3)	1.1812 (2)	0.31099 (5)	0.0237 (3)	
C8	0.1226 (3)	1.1921 (2)	0.36398 (6)	0.0235 (3)	
C9	0.2420 (2)	1.0224 (2)	0.40602 (5)	0.0189 (3)	
H9	0.1862	1.0290	0.4425	0.023*	0.73 (6)
F10	0.1593 (5)	1.0332 (5)	0.45796 (13)	0.0224 (10)	0.287 (5)
F10'	0.6956 (8)	0.6688 (8)	0.33028 (14)	0.0240 (13)	0.213 (5)
H1	0.258 (4)	0.605 (3)	0.4759 (7)	0.037 (5)*	
H6	0.456 (3)	0.995 (3)	0.2630 (7)	0.030 (4)*	
H7	0.114 (3)	1.296 (3)	0.2808 (7)	0.034 (4)*	
H8	−0.007 (3)	1.315 (3)	0.3730 (6)	0.030 (4)*	

### Atomic displacement parameters (Å^2^)

**Table:**

	*U*^11^	*U*^22^	*U*^33^	*U*^12^	*U*^13^	*U*^23^
N1	0.0108 (5)	0.0275 (6)	0.0168 (5)	0.0041 (4)	0.0032 (4)	0.0053 (4)
C2	0.0118 (6)	0.0233 (6)	0.0149 (5)	0.0003 (4)	0.0029 (4)	−0.0014 (4)
O3	0.0128 (5)	0.0416 (6)	0.0234 (5)	0.0072 (4)	0.0066 (3)	0.0104 (4)
C4	0.0112 (5)	0.0201 (6)	0.0170 (6)	−0.0023 (4)	0.0011 (4)	−0.0004 (4)
C5	0.0167 (6)	0.0225 (6)	0.0185 (6)	0.0016 (5)	0.0030 (5)	−0.0002 (5)
C6	0.0233 (7)	0.0274 (7)	0.0171 (6)	−0.0013 (5)	0.0028 (5)	0.0017 (5)
C7	0.0205 (6)	0.0231 (6)	0.0251 (6)	−0.0002 (5)	−0.0019 (5)	0.0073 (5)
C8	0.0192 (6)	0.0191 (6)	0.0324 (7)	0.0032 (5)	0.0052 (5)	0.0007 (5)
C9	0.0171 (6)	0.0202 (6)	0.0200 (6)	−0.0013 (5)	0.0050 (5)	−0.0017 (4)
F10	0.0267 (15)	0.0234 (14)	0.0195 (16)	0.0052 (10)	0.0104 (10)	−0.0014 (10)
F10'	0.026 (2)	0.029 (2)	0.0184 (18)	0.0116 (15)	0.0068 (14)	0.0018 (13)

### Geometric parameters (Å, °)

**Table:**

N1—C2	1.3354 (16)	C6—C7	1.3824 (19)
N1—N1^i^	1.384 (2)	C6—H6	0.989 (15)
N1—H1	0.84 (2)	C7—C8	1.3856 (19)
C2—O3	1.2315 (14)	C7—H7	0.971 (17)
C2—C4	1.4875 (16)	C8—C9	1.3822 (18)
C4—C9	1.3890 (17)	C8—H8	0.945 (17)
C4—C5	1.3916 (17)	C9—F10	1.373 (4)
C5—F10'	1.299 (5)	C9—H9	0.9600
C5—C6	1.3797 (18)	F10—H9	0.4136
C5—H5	0.9600	F10'—H5	0.3448
			
C2—N1—N1^i^	117.97 (12)	C5—C6—C7	119.69 (12)
C2—N1—H1	123.5 (11)	C5—C6—H6	119.3 (10)
N1^i^—N1—H1	116.7 (12)	C7—C6—H6	121.0 (10)
O3—C2—N1	121.31 (11)	C6—C7—C8	120.16 (12)
O3—C2—C4	121.94 (11)	C6—C7—H7	119.4 (10)
N1—C2—C4	116.75 (10)	C8—C7—H7	120.4 (10)
C9—C4—C5	118.28 (11)	C9—C8—C7	119.64 (12)
C9—C4—C2	123.46 (11)	C9—C8—H8	118.0 (9)
C5—C4—C2	118.20 (10)	C7—C8—H8	122.3 (9)
F10'—C5—C6	117.16 (18)	F10—C9—C8	118.83 (14)
F10'—C5—C4	121.64 (17)	F10—C9—C4	120.10 (14)
C6—C5—C4	121.15 (11)	C8—C9—C4	121.07 (11)
C6—C5—H5	119.4	C8—C9—H9	119.4
C4—C5—H5	119.5	C4—C9—H9	119.5
			
N1^i^—N1—C2—O3	−1.9 (2)	F10'—C5—C6—C7	178.7 (2)
N1^i^—N1—C2—C4	178.67 (12)	C4—C5—C6—C7	1.29 (19)
O3—C2—C4—C9	−147.25 (13)	C5—C6—C7—C8	−0.64 (19)
N1—C2—C4—C9	32.16 (16)	C6—C7—C8—C9	−0.29 (19)
O3—C2—C4—C5	29.97 (17)	C7—C8—C9—F10	−178.88 (16)
N1—C2—C4—C5	−150.62 (11)	C7—C8—C9—C4	0.61 (19)
C9—C4—C5—F10'	−178.3 (2)	C5—C4—C9—F10	179.49 (16)
C2—C4—C5—F10'	4.3 (3)	C2—C4—C9—F10	−3.3 (2)
C9—C4—C5—C6	−0.97 (18)	C5—C4—C9—C8	0.01 (18)
C2—C4—C5—C6	−178.33 (11)	C2—C4—C9—C8	177.23 (11)

Symmetry codes: (i) −*x*+1, −*y*+1, −*z*+1.

### Hydrogen-bond geometry (Å, °)

**Table:**

*D*—H···*A*	*D*—H	H···*A*	*D*···*A*	*D*—H···*A*
N1—H1···O3^ii^	0.84 (2)	2.05 (2)	2.8549 (16)	160.4 (16)
N1—H1···O3^i^	0.84 (2)	2.325 (16)	2.6302 (14)	101.8 (14)
C8—H8···O3^iii^	0.945 (17)	2.416 (16)	3.2687 (17)	150.0 (12)

Symmetry codes: (ii) *x*−1, *y*, *z*; (i) −*x*+1, −*y*+1, −*z*+1; (iii) *x*−1, *y*+1, *z*.

## References

[bb1] Ahmad, R., Iqbal, R., Akhtar, R. H., Haq, Z. U., Duddeck, H., Stefaniak, L. & Sitkowski, J. (2001). *Nucleosides Nucleotides Nucleic Acids*, **20**, 1671–1682. 10.1081/NCN-100105903 11580193

[bb2] Al-Soud, Y. A., Al-Deeri, M. N. & Al-Mosoudi, N. A. (2004). *Farmaco*, **59**, 775–783. 10.1016/j.farmac.2004.05.006 15474054

[bb3] Al-Talib, M., Tastoush, H. & Odeh, N. (1990). *Synth. Commun.* **20**, 1811–1814.

[bb4] Chopra, D., Mohan, T. P. & Vishalakshi, B. (2006). *Acta Cryst.* E**62**, o3085–o3086. 10.1107/S010827010602378X 16954636

[bb5] El-Emam, A. A., Al–Deeb, O. A., Al–Omar, M. & Lehmann, J. (2004). *Bioorg. Med. Chem.* **12**, 5107–5113. 10.1016/j.bmc.2004.07.033 15351394

[bb6] Oxford Diffraction (2002). *CrysAlis CCD* Oxford Diffraction, Wrocław, Poland.

[bb7] Oxford Diffraction (2006). *CrysAlis RED* Oxford Diffraction, Wrocław, Poland.

[bb8] Sheldrick, G. M. (2008). *Acta Cryst.* A**64**, 112–122. 10.1107/S0108767307043930 18156677

[bb9] Silva, J. F. P., Ellena, J., Ferreira, M. de L., Mascarenhas, Y. P., de Souza, M. V. N., Vasconcelos, T. R. A., Wardell, J. L. & Wardell, S. M. S. V. (2006). *J. Mol. Struct.* **788**, 63–71.

[bb10] Souza, M. V. N. de, Wardell, S. M. S. V., Wardell, J. L., Low, J. N. & Glidewell, C. (2007). *Acta Cryst.* E**63**, o230–o232. 10.1107/S0108270107002788 17339722

[bb11] Yousif, M. Y., Ismail, A. M., Elman, A. A. & El–Kerdawy, M. M. (1986). *J. Chem. Soc. Pak.* **8**, 183–187.

[bb12] Zareef, M. & Iqbal, R. (2007). *Phosphorus Sulfur Silicon Relat. Elem.* **182**, 281-298.

[bb13] Zheng, X., Li, Z., Wang, Y., Chen, W., Huang, Q., Liu, C. & Song, G. (2003). *J. Fluorine Chem.* **117**, 163–169.

